# Editorial: Immunity to Cytomegalovirus Infections: Challenges and Therapeutic Opportunities

**DOI:** 10.3389/fimmu.2022.889690

**Published:** 2022-03-28

**Authors:** Rebeca Alonso-Arias, Ramon Arens, Marco A. Moro-García

**Affiliations:** ^1^ Immunology Department, Hospital Universitario Central de Asturias, Oviedo, Spain; ^2^ Department of Cardiac Pathology, Health Research Institute of the Principality of Asturias (ISPA), Oviedo, Spain; ^3^ Laboratory Medicine Department, Hospital Universitario Central de Asturias, Oviedo, Spain; ^4^ Department of Immunology, Leiden University Medical Center, Leiden, Netherlands

**Keywords:** cytomegalovirus (CMV), inflammatory diseases, immunodeficiencies, immunosenescence, T lymphocytes

Human cytomegalovirus (CMV) is a double-stranded DNA virus belonging to the *Herpesviridea* family. Its transmission, through different fluids such as saliva, sexual contact, blood, and breast milk, makes its prevalence high, and the probability of infection increases with age. The implications of acute infection are of relevance for immunocompromised individuals (such as neonates and transplantation patients), and such CMV infections can lead to long lasting complications and even mortality. CMV is also of importance for the healthy population due to its chronicity and latency throughout the life of the infected individual. The development of an inflationary immune response over the years, which is not fully clarified, is a wide field of research where we can deepen our knowledge on the CMV-host interactions.

This Research Topic gives us a comprehensive overview of the current knowledge of CMV infection in various situations, as well as its possible solution or attenuation. This monograph includes thirteen articles: nine original articles and four review articles. The authors invited scientific collaborators to this collection based on their unique and specific findings on CMV under physiological and pathological conditions including: (a) mechanisms regulating CMV immune evasion, (b) the role of CMV in the alterations suffered by the immune system in patients with inflammatory chronic diseases, (c) CMV infection in the context of primary and secondary immunodeficiencies, (d) influence of CMV infection on the immunosenescence process associated to aging and (e) therapeutic opportunities in the management of CMV disease.

In the article by Reus et al., sex differences in the response of T lymphocytes to different CMV proteins are explored. The response of both CD4+ and CD8+ T lymphocytes was greater and more proinflammatory in men than in women. These findings may help understand sex differences in CMV-associated pathologies. Jackson et al. analyzed the effect of latent CMV infection on the secretome of CD14+ monocytes, finding an increase in the production of the chemokines CCL8 and CXCL10. The CD14+ latency-associated secretome also suppresses the anti-viral activity of stimulated CD4+ T cells. Moreover, co-culture of activated autologous CD4+ T cells with latently infected monocytes resulted in reactivation of CMV at levels comparable to those observed using M-CSF and Il-1b cytokines. This mechanism could be a strategy of CMV to reactivate and achieve local dissemination in peripheral tissues. Another article that studies the CMV latency period is the work by Griessl et al. showing that the IE, E and L genes are transcribed during latency, but in a stochastic manner, not following the IE-E-L cascade pattern. In addition, the transcripts that code for memory inflation-driving antigenic peptides rarely coincide with those that code for immune evasion proteins. This stochastic expression could explain why immune evasion is not operative in latently infected cells, however, it does not interfere with memory inflation. Another possible evasion mechanism studied in this monograph is the one analyzed by Zhang et al. where they observe that the expression of certain microRNAs that interact with IFN receptor 1 (IFNAR1) could achieve immune evasion, both in lytic and latent infection. van den Berg et al. investigated the impact of CMV infection on influenza-specific CD8+ T cells and the response to influenza infection in the elderly. CMV infection does not appear to diminish the influenza-specific response in acute CMV infection.

In the section on CMV infection associated with other pathologies, we find several very interesting works such as that of García-Torre et al. that relates the level of proinflammatory cytokines with the functional status of patients with chronic heart failure and with CMV infection. They found higher levels of all cytokines in patients with heart failure compared to healthy controls, as well as a direct correlation between levels of proinflammatory cytokines and worse functional status in patients. This cytokine production was much higher in CMV-infected patients, and anti-CMV antibodies correlated with levels of proinflammatory cytokines. The review presented by Alonso-Álvarez et al. tells us about the relationship between CMV infection and hematological tumors. The evolutionary symbiotic relationship between herpesviruses and humans disappears in immunosuppressed patients, especially in hematological patients. New procedures in transplantation have been introduced as well as new treatments to manipulate the composition of the graft and its functionality. In addition, new drugs have also been introduced to treat CMV infection. Another point discussed in this interesting review is the effects of CMV in terms of mortality or progression in patients with hematological tumors treated with immunochemotherapy or new molecules or in patients who have received SCT. El Baba and Herbein review the immunological profile in cancer patients infected by CMV. The changes promoted by CMV infection are related to immunosenescence and phenotypes that lead to immunosuppressive tumor environments and oncomodulation. CMV-induced evasion mechanisms play a major role in developing new approaches in tailored therapeutics against CMV, especially since immunotherapy has revolutionized therapeutic strategies against cancer.

Luo et al. discuss CMV infection and CMV-specific immune reconstitution in the context of haploidentical stem cell transplantation (SCT). The cure in haploidentical stem cell transplantation (haploSCT) is seriously hampered by CMV infection and delayed immune reconstitution compared to HLA-Matched stem cell transplantation. The authors provided an update on CMV infection and CMV-specific immune recovery in this fast-evolving field. Another very interesting original work is the one carried out by Gergely et al. where therapeutic vaccination against CMV in hematopoietic cell transplantation (HCT) recipients is reviewed. Therapeutic vaccination aims at restimulation and expansion of specific transferred CD8+ T lymphocytes in the recipient of HCT. Their preclinical research data provide an argument for using pre-emptive therapeutic vaccination to improve antiviral protection by adoptive cell transfer in HCT recipients with diagnosed CMV reactivation. In the review written by García-Ríos et al. the possibilities of using CMV-specific T-cell adoptive transfer as a treatment against CMV infection in solid organ transplantation (SOT) recipients are described. This option may be a therapeutic alternative to reconstitute the specific T cell response against CMV and control CMV viremia in SOT patients.

Pardieck et al. declare in their article that a vaccine that induces immunity against CMV in an immunocompromised or immunoimmature population is highly needed. Current insight encourages that a protective immune response to CMV might benefit from the induction of virus-specific T cells. The combination of antibodies and CD8+ T cell-eliciting vaccines provides a collaborative improvement of humoral and cellular immunity allowing to improve protection against CMV. In the article by Šustić et al. the role of memory CD8+ T cells, generated by a CMV vaccine vector expressing NKG2D ligand, with an effector-like phenotype and distinct functional features is studied. CMV is an attractive vaccine vector due to its large genome with few non-essential genes that can be easily manipulated. Their results shed new insights into the phenotypical and functional distinctness of memory CD8+ T cells induced with CMV vectors expressing cellular ligands for the NKG2D receptor.

We hope that all articles in this Frontiers in Immunology Research Topic will serve to broaden the knowledge of researchers and clinicians in the field of CMV infection. In addition, we hope that these works will serve as a stimulus to expand research on the mechanisms behind infection by this virus that will result in an improvement in the lives of patients.

## Author Contributions

MM-G wrote the editorial. RA-A and RA reviewed and corrected the editorial. All authors contributed to the article and approved the submitted version.

## Conflict of Interest

The authors declare that the research was conducted in the absence of any commercial or financial relationships that could be construed as a potential conflict of interest.

## Publisher’s Note

All claims expressed in this article are solely those of the authors and do not necessarily represent those of their affiliated organizations, or those of the publisher, the editors and the reviewers. Any product that may be evaluated in this article, or claim that may be made by its manufacturer, is not guaranteed or endorsed by the publisher.

